# A Comprehensive Review of Artificial Intelligence for Brain Tumor Analysis: Taxonomy, Robustness, and Open Challenges in Neuro-Oncology

**DOI:** 10.3390/jimaging12060228

**Published:** 2026-05-27

**Authors:** Mais Haj Qasem, Thamer Mitib Al Sariera, Khadija Alhumaid, Shadi Majed Alshraah, Ahmad Subhi Salem Mufleh, Naceur Chihaoui

**Affiliations:** 1 Department of Data Science and Artificial Intelligence, Amman Arab University, Amman 11953, Jordan; m.hajqasem@aau.edu.jo; 2 Department of Computer Science, Amman Arab University, Amman 11953, Jordan; t.alsariera@aau.edu.jo; 3 Research & Innovation Division, Rabdan Academy, Abu Dhabi 114646, United Arab Emirates; kalhumaid@ra.ac.ae; 4 English Department, Preparatory Year Deanship, Prince Sattam bin Abdulaziz University, Al-Kharj 11942, Saudi Arabia; 5 Basic Science Department, Preparatory Year Deanship, Prince Sattam bin Abdulaziz University, Al-Kharj 11942, Saudi Arabia; a.mufleh@psau.edu.sa (A.S.S.M.); n.chihaoui@psau.edu (N.C.)

**Keywords:** brain tumor detection, artificial intelligence, deep learning, explainable AI, generative AI, diffusion models, federated learning, multimodal learning, precision neuro-oncology, medical imaging

## Abstract

Detecting brain tumors can be challenging as a clinical problem because of tumor heterogeneity and reliance on manual neuroimaging interpretation, which can be prone to human error. Artificial intelligence (AI) has shown strong potential as a clinical decision-support tool, assisting radiologists in improving diagnostic accuracy and supporting the interpretation of neuroimaging data. AI using machine learning (ML) and deep learning (DL) algorithms has performed credibly in tumor detection, segmentation, and classification tasks. Challenges such as dataset bias, limited generalization, lack of explainability, and high computational costs must be addressed before clinical application. This article provides a comprehensive review of AI methods applied to brain tumor imaging, with a primary focus on adult diffuse gliomas and secondary coverage of brain metastases, meningiomas, and pediatric tumors where relevant. The major contribution of this review is a new three-factor (diagnostic tasks, learning strategies, and data modalities) taxonomy. Beyond accuracy-based metrics, we provide a qualitative assessment of robustness, generalization, and the principal barriers to clinical adoption identified in the published literature, while acknowledging that comprehensive clinical utility evidence remains an open research direction.

## 1. Introduction

Brain tumors pose a major challenge in neuro-oncology because of their heterogeneous appearance, biological behavior, and complex anatomical location, which complicate the diagnosis and treatment planning [1]. Manual analysis is time-consuming and subject to inter-observer variability, particularly in small lesions with infiltrative margins and tumors in complex brain regions [2,3]. Thus, an important clinical goal is to obtain more accurate diagnoses of this disease.

In recent years, AI—particularly ML and DL—has demonstrated strong potential for automating brain tumor analysis tasks, such as detection, segmentation, and classification [4]. CNNs and their variants have achieved high performance on benchmark datasets, often matching expert-level accuracy [5,6,7]. More recently, hybrid models based on transformer architectures have further enhanced feature representation and multimodal learning through imaging data supplemented with clinical and molecular information [8]. These advancements have enabled AI to extract high-dimensional patterns from neuroimaging data that are not easily perceptible to the human eye.

Despite these advances, the translation of AI models into routine clinical practice remains challenging. One major challenge is the reliance on curated benchmark datasets, such as BraTS. These datasets do not sufficiently represent real-world clinical variability, which causes poor generalization across different scanners and institutions. Moreover, numerous deep learning models operate as black boxes, limiting their explanations and reducing clinician trust in critical decision-making [9]. In addition, regulations on data protection obstruct centralized sharing of data, and the cost-prohibitive training of large-scale models limits their use in clinical settings [10,11].

Current research mainly measures the performance of algorithms, typically using accuracy-based metrics. Taha et al. [5] have carried out a systematic review on machine learning vs deep-learning for brain-tumor detection, for example. Khalighi et al. [3] presented the latest advances in AI for applications in neuro-oncology disease diagnosis, prognosis, and precision treatment. Nevertheless, studies on AI algorithms emphasize performance metrics but mainly overlook many other characteristics such as clinical readiness, interpretability, data privacy, and real-world deployment [12].

In contrast to existing surveys, this study provides a clinically oriented synthesis of AI techniques for brain-tumor analysis. Unlike prior reviews that mainly focus on algorithmic performance, this survey introduces a unified taxonomy that integrates diagnostic tasks, learning paradigms, and data modalities to enable a more comprehensive understanding of its clinical applicability. In addition, this study moves beyond accuracy-based evaluations. This study also delves into recent developments in explainable AI (XAI), generative diffusion models for the synthesis of medical images, federated learning that preserves privacy in training, and multimodal fusion techniques that blend imaging with molecular information. We conclude by describing the important challenges and laying a research roadmap towards safe, interpretable, and scalable AI systems for the clinical diagnosis of brain tumors.

Scope and clinical focus. While the AI methods discussed in this review are broadly applicable to multiple brain tumor entities, the primary clinical focus is placed on adult diffuse gliomas, the most extensively studied entity in the AI neuro-oncology literature and the dominant target of public benchmarks such as BraTS. Secondary coverage is provided for brain metastases, meningiomas, and pediatric tumors, where representative AI evidence exists. With respect to the diagnostic pipeline, this review addresses preoperative diagnosis, tumor segmentation, molecular classification, and post-treatment response monitoring of brain tumors. Detailed coverage of radiotherapy treatment planning, dose optimization, and surgical robotics is considered outside the primary scope, although relevant intersections are noted where appropriate in the text. This focused scope allows the proposed taxonomy and qualitative robustness assessment to remain anchored to a clinically coherent set of tasks, rather than encompassing every possible neuro-oncology application.

This survey is organized as follows to address the above issues and research gaps: Section 2 provides a clinical context by describing the main brain tumor types and multidisciplinary clinical decision-making pathway. Section 3 outlines how the selected studies were identified and assessed in this review. The proposed taxonomy is introduced in Section 4 of this paper. The model performance is evaluated beyond accuracy in Section 5, including robustness and clinical readiness. Section 6, Section 7, Section 8 and Section 9 cover XAI, generative models, federated learning, and multimodal approaches. We discuss the computational efficiency, complications of real-world deployment, and future work in Section 10, Section 11 and Section 12. Finally, this survey has limitations, which are detailed in Section 13.

## 2. Brain Tumor Types and Clinical Decision-Making Pathways

This integrated diagnostic framework has direct implications for AI-based analysis, as models trained solely on histological imaging may fail to capture the molecular heterogeneity that now defines clinical classification. Understanding the key tumor entities within this framework is therefore essential before examining how AI methods are applied to each.

### 2.1. Tumor Heterogeneity and WHO CNS5 Classification

Brain tumors represent a heterogeneous group of neoplasms that differ substantially in terms of histological origin, molecular profile, clinical behavior, and prognosis. The 2021 WHO Classification of Tumors of the Central Nervous System (CNS5) marked a paradigm shift by requiring an integrated diagnosis based on both histological and molecular features, moving beyond morphology alone.

The most clinically significant brain tumor types include diffuse gliomas (IDH-mutant and IDH-wildtype glioblastoma), meningiomas, brain metastases from systemic cancers, medulloblastomas, and other pediatric embryonal tumors. These tumor types differ fundamentally in terms of their biology and prognosis. IDH-mutant gliomas have a significantly better prognosis and respond differently to treatment compared to IDH-wildtype glioblastoma (GBM), which remains the most aggressive primary brain tumor with a median survival of approximately 15 months despite standard-of-care therapy. Pediatric diffuse gliomas, such as H3K27M-altered diffuse midline glioma (DIPG), are biologically distinct entities and not simply smaller adult tumors, and require entirely different therapeutic approaches.

### 2.2. Clinical Presentation and Initial Neuroimaging

**Figure 1:**
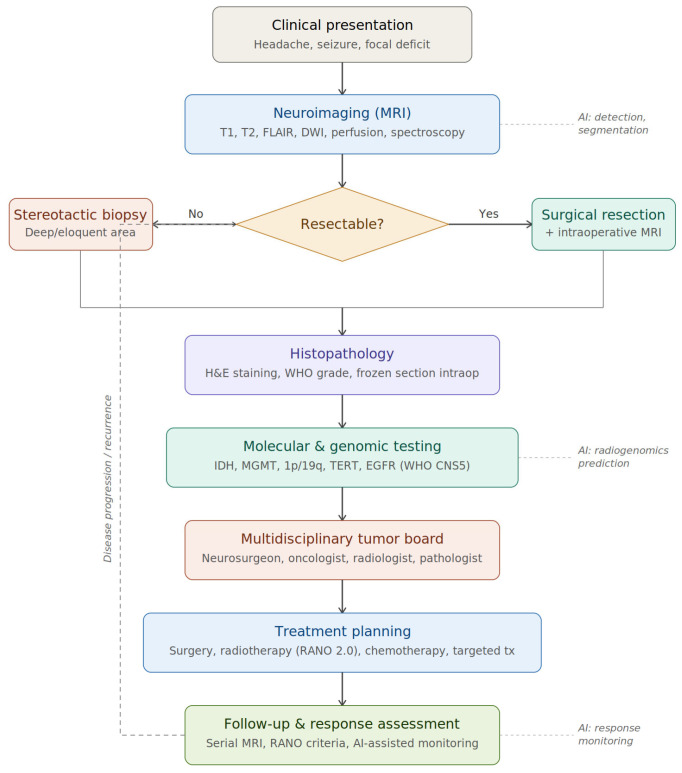
Clinical decision-making pathway in neuro-oncology, from initial presentation and neuroimaging to histopathological and molecular diagnosis, multidisciplinary tumor-board review, treatment planning, and longitudinal follow-up. The AI integration points reviewed in this survey are annotated along the pathway.

The diagnostic process typically begins with clinical presentation: headache, seizures, or focal neurological deficits prompt referral for neuroimaging studies. Multisequence MRI remains the cornerstone of the initial evaluation, incorporating T1-weighted, T2/FLAIR, diffusion-weighted imaging (DWI), perfusion MRI, and MR spectroscopy sequences. These provide complementary information on tumor location, extent, vascularity, and metabolic activity, which collectively inform the differential diagnosis. The complete clinical decision-making pathway is illustrated in Figure 1.

### 2.3. Surgical Decision-Making and Intraoperative Assessment

The surgical strategy depends on the lesion location, resectability, and patient’s functional status. For accessible lesions, maximal safe resection is pursued, often aided by intraoperative MRI, fluorescence-guided surgery (5-ALA), and frozen-section histopathological assessment for real-time diagnosis. For lesions in deep or eloquent brain regions, a stereotactic biopsy is performed to obtain tissue while minimizing neurological risk.

### 2.4. Histopathology and Molecular Testing

Resected or biopsied tissues undergo formal histopathological examination, including hematoxylin and eosin (H&E) staining and immunohistochemistry, followed by comprehensive molecular and genomic profiling. Key molecular markers required for the WHO CNS5 integrated diagnosis include IDH mutation status, MGMT promoter methylation, 1p/19q codeletion, TERT promoter mutations, and EGFR amplification. These markers are critical for diagnosis and therapy. For instance, MGMT methylation predicts response to temozolomide chemotherapy in GBM, directly influencing treatment selection.

### 2.5. Multidisciplinary Tumor Board and Treatment Planning

Integrated findings are reviewed by a multidisciplinary tumor board comprising neurosurgeons, neuro-oncologists, radiation oncologists, neuroradiologists, and neuropathologists. Treatment is individualized based on tumor type, molecular profile, and patient factors, and may include radiotherapy (guided by RANO 2.0 criteria), chemotherapy, or targeted molecular therapy.

### 2.6. Follow-Up and Response Assessment

Post-treatment follow-up relies on serial MRI interpreted using standardized response assessment criteria—RANO and AI-RANO—to monitor treatment response, pseudo-progression, and recurrence. This phase represents an emerging area for AI-assisted monitoring, where automated longitudinal analyses can support timely clinical decision-making.

This clinical complexity underscores the importance of AI systems designed and validated within specific, well-defined clinical contexts, and not evaluated solely on generic benchmark accuracy. The clinical pathway depicted in Figure 1 serves as a real-world framework against which all AI contributions reviewed in this survey are assessed.

## 3. Methodology of Literature Review

This study adopts a structured review approach to systematically analyze the recent contributions of artificial intelligence (AI) to brain tumor analysis. Relevant studies were retrieved from major academic databases, including IEEE Xplore, PubMed, Scopus, and Google Scholar.

The literature search combined three concept groups using Boolean operators: (i) tumor target—“brain tumor” OR “glioma” OR “glioblastoma” OR “meningioma” OR “brain metastasis” OR “pediatric brain tumor”; (ii) AI methodology—“artificial intelligence” OR “machine learning” OR “deep learning” OR “convolutional neural network” OR “transformer” OR “federated learning” OR “explainable AI” OR “generative model” OR “diffusion model”; and (iii) clinical task—“detection” OR “classification” OR “segmentation” OR “radiogenomics” OR “survival prediction” OR “treatment response” OR “medical imaging” OR “MRI.” Database-specific adaptations were applied: PubMed used MeSH terms (“Brain Neoplasms”[Mesh] AND “Artificial Intelligence”[Mesh]); IEEE Xplore and Scopus used title/abstract/keyword fields; Google Scholar was used for citation chaining and to capture preprints from arXiv that were subsequently published in peer-reviewed journals. The literature search will be conducted between January and March 2025 and updated in June 2025 to include the most recent publications.

To ensure comprehensive coverage of recent developments, studies published between January 2018 and June 2025 were included. The inclusion criteria were as follows: (i) peer-reviewed journal articles or full conference proceedings; (ii) AI/ML/DL methods applied to brain tumor analysis (detection, segmentation, classification, radiogenomics, prognosis, or response assessment); (iii) reporting of a quantitative evaluation of at least one neuroimaging dataset; and (iv) full text available in English. The exclusion criteria were as follows: (i) studies on non-brain tumors or non-neuroimaging applications; (ii) abstract-only conference posters without methodological details; (iii) editorials, letters, and case reports without algorithmic contribution; (iv) duplicate publications of the same study; and (v) preprints not subsequently published in peer-reviewed venues. The retained studies form the basis for the narrative synthesis presented in the subsequent sections, with a lightweight bibliometric overview provided in Section Bibliometric Overview of the Reviewed Literature. We note that this work is presented as a structured narrative review with an approximate bibliometric summary, rather than as a formal systematic review or scientometric study; this scope choice and its implications are explicitly acknowledged in Section 13.1 of this article.

After removing duplicate and irrelevant studies, the remaining articles were carefully analyzed and categorized according to the proposed taxonomy, which includes diagnostic tasks, learning paradigms and data modalities. Particular emphasis was placed on studies related to explainable artificial intelligence (XAI), federated learning, generative models, and multimodal data integration.

**Figure 2:**
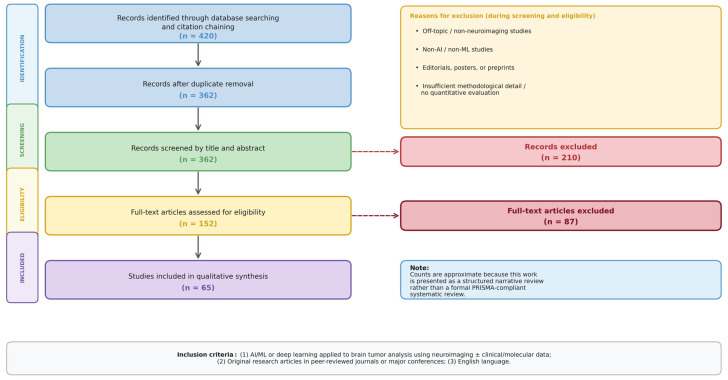
PRISMA-inspired literature search and study-selection workflow was used in this structured narrative review. The study counts are approximate because the review is not presented as a formal PRISMA-compliant systematic review.

The study selection process followed a PRISMA-inspired approach, as illustrated in Figure 2, without stage-by-stage counts (see Section 13.1 for scope).

### 3.1. Bibliometric Overview of the Reviewed Literature

To provide a structured quantitative context for the surveyed literature, the reviewed studies were grouped according to five descriptive categories: (i) diagnostic task (segmentation, classification, detection, radiogenomics or molecular prediction, survival prediction, treatment-response assessment, and post-treatment MRI monitoring); (ii) tumor entity (adult diffuse glioma and glioblastoma, brain metastases, meningioma, and pediatric brain tumors); (iii) validation strategy (internal hold-out split, multi-center, external validation, and prospective evaluation); (iv) study design (retrospective versus prospective); and (v) data source (public benchmarks such as the BraTS challenge series, TCIA-derived cohorts, and single-institution clinical datasets). This categorization is presented as an approximate overview to characterize the field; it does not constitute a formal scientometric or systematic review, the limitations of which are acknowledged in Section 13.1.

**Figure 3:**
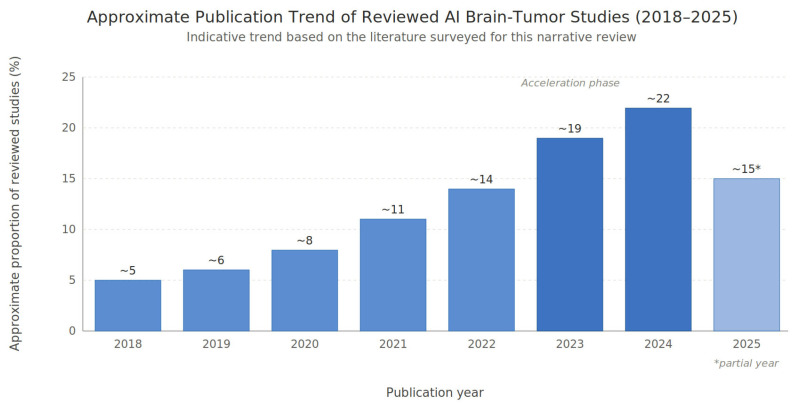
Approximate publication trend of the reviewed AI brain-tumor studies between 2018 and 2025. The lighter bar for 2025 reflects partial year coverage at the time of writing.

Table 1 summarizes the approximate distribution of the reviewed studies across these categories, with brief observations accompanying each entry. Figure 3 visualizes the corresponding publication trend across the 2018–2025 window.

Several patterns emerged from this overview. Most of the reviewed works target segmentation, classification, and detection, while prognostic and response-assessment tasks remain comparatively underrepresented. Adult diffuse glioma is the dominant tumor entity, while pediatric tumors, brain metastases, and meningioma have been addressed by a limited number of studies despite their clinical importance. The validation profile is consistent with broader observations in medical AI: most studies rely on a single dataset or single institution, a limited proportion report formal external validation, and prospective evaluation embedded in a real clinical workflow is very rare. Public benchmarks, chiefly the BraTS family, with growing contributions from BraTS-PEDs [13] and the 2024 post-treatment glioma challenge [14], underpin most reported results, which reinforces both the comparability of methods and the field’s benchmark dependency. Complementary cohorts, such as UCSF-PDGM [15] and Burdenko-GBM-Progression datasets [16], are increasingly used to assess generalization, often with substantial cross-institution performance degradation [17]. Scanner and vendor diversity remains restricted across most reviewed cohorts.

Taken together, these observations highlight three translational concerns that recur throughout the remainder of this review: a clinical-maturity gap between accuracy-centric benchmark performance and the demands of clinical deployment; a benchmark dependency that may overstate generalization to unseen scanners, vendors, and patient populations; and a lack of prospective multicenter evaluation with clinically meaningful endpoints. These concerns motivated the qualitative robustness analysis presented in Section 5 and the deployment gap discussion in Section 11. The proportions reported here are approximate, and a formal systematic review with a quantitative bibliometric analysis is acknowledged as a valuable line of future work in Section 13.1.

## 4. A Taxonomy of AI Techniques for Brain Tumor Analysis

Most previous survey studies on AI for brain tumor analysis have typically classified the methods into categories of algorithm types, imaging methods, or specific clinical tasks. Although these classifications provide insights into the model’s performance, they investigate these dimensions independently. Consequently, the connection between diagnostic goals, pedagogical approaches, and data characteristics is currently under-researched in a single framework.

Over the last decade, AI studies for brain tumor analysis have witnessed exponential growth, resulting in a plethora of methods addressing various clinical objectives, learning paradigms, and data representations. Surveys of this kind have been published to review the algorithmic performance in several studies. However, some do not cover the organizational perspective. Many such surveys do not link diagnostic tasks, methodological choices, and data modalities to clinical applicability. To overcome this limitation, we present a structured taxonomy that organizes existing AI-based studies along three complementary dimensions: (i) diagnostic task, (ii) learning methodology, and (iii) data modality. This classification identifies and synthesizes current trends in the field, with gaps relevant to precision neuro-oncology.

**Figure 4:**
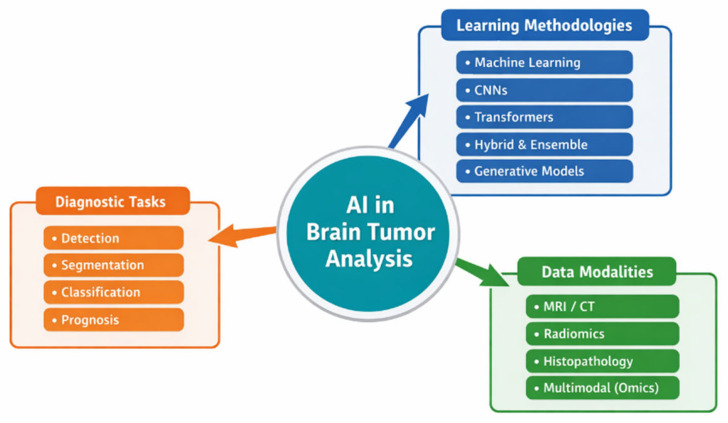
Proposed taxonomy of AI techniques for brain-tumor analysis organized by diagnostic tasks, learning methodologies and data modalities.

Figure 4 illustrates the proposed taxonomy, which classifies AI-based studies on brain tumors according to diagnostic tasks, learning methods, and data modalities.

In clinical practice, most AI applications focus on tumor detection and classification because of the greater availability of labeled imaging datasets and well-defined evaluation metrics. In a study by Mahmud et al. [6], MRI scans were processed by deep CNNs with high accuracy, showing the effectiveness of end-to-end deep learning pipelines in tumor region detection. Similarly, Taha et al.’s [5] systematic review showed that detection and classification tasks are the dominant contributions in the literature and often outperform traditional machine learning techniques on benchmark datasets.

Tumor segmentation is more complex because it involves marking the edges and regions of the tumor, which is useful for surgical planning and radiotherapy. Surveys in neuroradiology indicate that segmentation models, while achieving strong benchmark results, are sensitive to imaging variability and specific scanner characteristics, which limits their robustness [7].

However, survival prediction and treatment response prognosis are still not well represented. According to Khalighi et al. [3], the lack of longitudinal datasets and standardized labels of outcomes still hampers the replacement field, which is clinically relevant. The findings indicate that the prioritization of current research projects is dictated more by the availability of data than by clinical needs that would benefit from the highest impact.

### 4.1. Method-Oriented Taxonomy

Brain tumor analysis methodologies are gradually transitioning from classical machine learning models to complex deep-learning models. The machine learning classifier combined with radiomics-based pipelines involves interpretable quantitative features, as per the review of Pacchiano et al. [2]. In addition, they are heavily reliant on handcrafted descriptors and careful feature selection.

Deep learning approaches, particularly CNN-based architectures, have become the dominant paradigm owing to their ability to learn hierarchical image representations directly from raw imaging data. Studies summarized in [5,6] consistently report the superior performance of CNNs compared to traditional machine learning methods on curated datasets, particularly in terms of accuracy, sensitivity, specificity, and F1-score for tumor detection and classification tasks.

Recently introduced advanced architectures aim to mitigate these limitations. Hollon et al. [8] showed that AI systems can use imaging and molecular information to classify gliomas. This indicates a transition to more complex and clinically useful modeling. Nevertheless, neuroradiology evaluations indicate that computationally heavy and less interpretable complex architectures may not be feasible for deployment [7]. Owing to such methodological challenges, interest in XAI [9] and efficiency-aware model design [11] is growing, as discussed in the following sections.

### 4.2. Data and Modality-Oriented Taxonomy

The third dimension of our taxonomy is the type of data that developers use to test their AI with and train their AI. Structural MRI is currently the most widely used method across all tasks because of its high soft-tissue contrast and extensive use in clinics. Recent studies have explored advanced deep learning architectures for brain MRI analysis, demonstrating improved tumor detection and segmentation performance in neuroimaging tasks [18]. Radiomics-based approaches allow the extraction of quantitative features from MRI data, which may support classical or hybrid learning pipelines. These approaches are better suited for interpretability, albeit at the cost of more complex preprocessing requirements [2].

The understanding of the true performance of models in real-world clinical environments is further limited by the reliance on a small number of curated benchmark datasets. As demonstrated in [19], evaluation metrics obtained from highly curated neuroimaging benchmarks may overestimate the true generalization capability of AI models. Empirical studies have shown that models trained on data from one institution often experience performance degradation when applied to external clinical datasets because of domain shift and dataset bias. Similarly, benchmarking bias may hinder the translational impact of AI systems when models are optimized primarily for benchmark performance rather than for clinical robustness [20].

Recent frameworks have used molecular, genomic, and clinical data together with imaging for precision neuro-oncology. Despite the strong potential of multimodal approaches, they face challenges related to data variation, privacy restrictions, and scale. This has raised interest in federated learning paradigms [10], which are discussed in the next sections.

Table 2 presents the differences associated with diagnostic tasks, learning schemes, and data types organized within the proposed taxonomy, along with representative studies. The table shows that detection and classification objectives predominate, CNN-based architectures are the most common, and curated benchmark datasets are heavily relied upon. Consequently, the study reveals the limited clinical maturity of prognosis-oriented and multimodal approaches, emphasizing the gap between performance reported at the benchmark and performance in real-world clinical settings. These findings highlight the need for evaluation methods that go beyond accuracy-based metrics, as further analyzed in the next section.

Further analysis based on taxonomy revealed several important findings. The predominance of tasks focused on detection and classification suggests that researchers prefer problems that are accompanied by large amounts of data. However, tasks focused on prognosis modeling have not received much attention owing to their complexities. Furthermore, it requires longitudinal and multimodal data. In addition, although CNN-based architectures are the dominant systems, there is an undeniable growing trend toward more sophisticated models such as multimodal and hybrid architectures. The latter combines CNN feature extraction with a transformer-based attention mechanism to achieve high accuracy. Research has also been conducted on the use of Vision Transformer (ViT)-based models for medical image analysis [21], showing that they can explore long-range connections more easily.

Despite these advancements, most studies still rely on benchmark imaging datasets, leading to concerns about generalization and robustness, which are limited by clinical diversity. The reliance of a model on its parameters affects the difference between the performance in experiments and clinical use. Medical-grade clinical trials for the highest-risk AI algorithms would require additional robustness, interpretability, and clinical readiness standards beyond mere accuracy.

## 5. Performance Analysis Beyond Accuracy

The most popular means of assessing the performance of an AI model developed for brain tumor analysis (BTA) is the overall accuracy and dice scores which may include (AUC). While these measurements can be helpful and informative, they typically only record a narrow slice of performance and miss clinically relevant failure modes, such as false negatives, localization errors, and sensitivity to distributional shifts. The use of these limitations in safety-critical medical settings can lead to erroneous conclusions regarding real-world usability. In other words, this subsection goes beyond an accuracy evaluation to offer a critical assessment of robustness, generalization, and clinical readiness on different datasets and deployment scenarios.

### 5.1. Limitations of Accuracy-Centric Evaluation

Metrics that focus only on accuracy tend to neglect important characteristics of model behavior, particularly in imbalanced datasets. For instance, in such datasets, the tumor regions occupy a small volume of an image. Studies have shown that high-accuracy models can be poorly calibrated and suffer from miscalibrated confidence estimates, causing them to be overconfident and incorrect [22,23]. Furthermore, typical segmentation metrics, such as the Dice score, fail to capture boundary accuracy or clinical relevance in cases of infiltration or ambiguity of tumor margins.

Recent studies in medical imaging indicate that assessment procedures must include further metrics, such as the sensitivity–specificity trade-off, uncertainty estimation, and clinically relevant error analysis [24]. If there is no such consideration, the model performance on benchmark datasets may not lead to trustworthy clinical decision support.

### 5.2. Robustness and Generalization Across Datasets

A key challenge for clinical deployment is the lack of robustness and generalization across imaging centers, scanners, and patient populations. Several AI models have been trained and evaluated on curated benchmark datasets, namely BraTS, where acquisition protocols and labeling standards tend to be more homogeneous. Research on data from other datasets showed that changing the dataset caused a dip in performance. It showed external cohort model performance degradation due to domain shift [20,25].

External validation is rarely performed, and when it is performed, a major drop in sensitivity and segmentation ability is observed. According to the findings, benchmark-driven evaluation can vastly overestimate real-world generalization capability, and hence restrict translational impact [26]. To structure the discussion of robustness and generalization, Table 3 organizes representative AI brain tumor studies along the dimensions most relevant to clinical translation: clinical task, dataset type, external validation status, reported generalization issues, and key translational limitations. This table is intended as a structured qualitative synthesis of the robustness and external validation characteristics reported across representative studies rather than a formal quantitative meta-analysis. Quantitative metrics (Dice, AUROC, confidence intervals, performance drops) were not uniformly reported across the cited sources and are therefore not tabulated; instead, we used concrete translational descriptors that can be traced to the characteristics described in each cited study.

Four translational patterns emerged consistently across the entries in Table 3. First, external validation remains the exception rather than the rule; most representative studies either do not report any external validation or do so only sporadically. Second, benchmark dependence is pervasive: a substantial share of the surveyed work trains and evaluates the same public benchmark family, leaving cross-institution generalization unverified. Third, scanner and vendor diversity is insufficient in most studies; protocol heterogeneity, field strength differences, and acquisition-vendor variability are rarely controlled, even though they are known sources of clinical performance degradation. Fourth, prospective clinical evaluation is essentially absent across the cited sources, which constrains what can be responsibly claimed about clinical readiness. These patterns are qualitative rather than meta-analytic and reflect what is reported in the cited primary sources. However, their consistency across tasks and tumor entities is sufficient to motivate the deployment gap analysis presented in Section 11.

This comparison demonstrates a substantial gap between benchmark and real-world results. Models that perform well in controlled experiments may not perform equally well on heterogeneous clinical data. Safety and dependability in deployment require assessment protocols that explicitly target cross-domain generalization, which in turn improves the reliability of clinical systems.

### 5.3. Clinical Readiness and Real-World Applicability

In addition to robustness, the practical deployability of the algorithm must be evaluated. Some factors that affect AI implementation in clinical trials include integration into clinical workflows, inference latency, and robustness. Research addressing real-world deployment highlights that impressive offline performance does not ensure safety during clinical use or efficacy [10,27].

In addition, acquisition protocols, patient populations, and institutional practices vary and can dramatically influence model behavior. Consequently, research-rated models have often stalled at the proof-of-concept stage. To properly tackle these challenges, assessment frameworks are required to measure both clinical feasibility and algorithmic performance.

**Figure 5:**
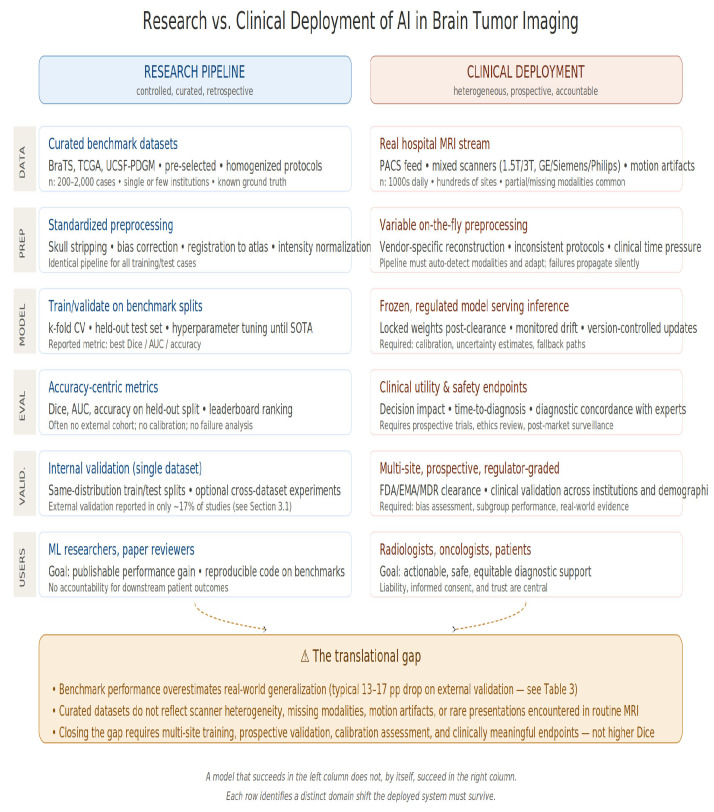
Comparison between research and clinical AI deployment settings across six dimensions: data, preprocessing, model behavior, evaluation, validation, and stakeholders. The figure highlights the translational gap between benchmark-oriented AI development and real-world clinical deployment.

To make explicit what changes when an AI model leaves a benchmark and enters a hospital, we contrast the two settings in Figure 5. The figure is organized as a six-row comparison rather than a single architecture diagram: each row (data, preprocessing, model, evaluation, validation, and stakeholders) shows where the research and clinical settings diverge, and the bottom bar summarizes the resulting translational gap that any deployed system must close.

Reading Figure 5 row by row reveals the divergence between research and clinical AI. At the data level, research uses a small set of curated cohorts (BraTS, TCGA, UCSF-PDGM) with known ground truth, whereas the clinical environment receives a continuous stream of MRI from heterogeneous scanners and protocols with partial or missing modalities. At the preprocessing and model levels, research can apply identical pipelines and tune hyperparameters to maximize a benchmark metric, whereas a clinically deployed model must run frozen weights through variables on-the-fly preprocessing while monitoring drift and failure modes. At the evaluation and validation levels, research typically reports the Dice coefficient, AUC, or accuracy on a held-out split, whereas clinical deployment requires multisite prospective validation, calibration, and clinically meaningful endpoints, such as decision impact and time-to-diagnosis. Finally, the user and accountability profile changes completely: ML researchers are accountable to peer reviewers, whereas clinicians, patients, and regulators bear the consequences of the model’s behavior.

Clinical AI must operate on heterogeneous data collected from different scanners, acquisition protocols, and patient populations, which often leads to significant performance degradation compared to curated benchmark datasets [19,20]. Several empirical studies have demonstrated that models trained on benchmark datasets, such as BraTS, may experience notable performance drops when evaluated on external clinical cohorts owing to domain shifts and dataset bias [20].

For clinical readiness, strong predictive performance, robustness to domain shifts, operational stability, and integration into the clinical decision-making process are required. Therefore, the demand for explainable and trustworthy AI approaches is discussed in the next section.

### 5.4. Summary and Link to Explainable AI

In conclusion, accuracy-centric metrics hide shortcomings in terms of robustness, generalization, and clinical relevance. The evidence reviewed in this section reveals that evaluation strategies should be designed with domain shift and uncertainty in mind, alongside deployment constraints. The above-mentioned reasons have motivated the adoption of explainable and trustworthy AI approaches, which we evaluate in the next section.

## 6. Explainable Artificial Intelligence (XAI) for Brain Tumor Diagnosis

The increased complexity of deep learning models has reduced their interpretability, which is a recognized concern for safe deployment in clinical settings. Brain tumor diagnosis depends on heterogeneous data sources, whose individual contributions to a model’s decision are often opaque. Explainable AI (XAI) methods aim to make certain aspects of model behavior inspectable. However, it is important to position XAI accurately at the outset of this section: XAI is a tool for debugging, sanity-checking, and clinical communication. XAI is not, by itself, evidence that a model is clinically valid, well-calibrated, robust, or safe to deploy. The remainder of this section reviews the principal XAI families used in brain tumor imaging, examines their known failure modes, and clarifies the boundary between what XAI can and cannot do for clinical translation.

Recent studies have reported that explainable AI methods can provide clinicians with a partial view of model behavior; however, this transparency should not be conflated with evidence of clinical reliability, which depends on independent validation pathways, as discussed in Section 6.3 [28].

### 6.1. Why Explainability Is Critical in Brain Tumor Diagnosis

Unlike traditional rule-based decision support tools, AI brain tumor models often do not expose the rationale for their predictions. Clinicians benefit from understanding not only the recommendations generated by a system but also the input features it relies on. However, interpretability alone does not establish clinical validity; a plausible explanation can accompany a poorly calibrated, biased, or unsafe model. Predictive accuracy and interpretability do not justify clinical use unless the model has undergone external validation, calibration assessment, and clinical-utility evaluation [29].

The most defensible role of XAI is debugging and sanity checking. Explanation methods can help identify model failure modes, dataset bias, and spurious correlations; for example, by revealing whether a model’s decision is driven by clinically relevant tumor regions or by unrelated artifacts such as scanner watermarks, motion artifacts, or skull-stripping residues. In this role, XAI reduces the risk of unsafe deployment by surfacing problems that aggregate accuracy metrics cannot detect. However, this does not prove that the model will generalize across institutions or that its outputs are clinically actionable.

### 6.2. Model-Agnostic and Model-Specific XAI Techniques

XAI approaches can be divided into model-agnostic and model-specific methods. Methods that can be applied to any model, regardless of the predictive algorithm, are called model-agnostic methods. For example, Local Interpretable Model Agnostic Explanations (LIME) and SHapley Additive exPlanations (SHAP). Model-agnostic methods can be used with any predictive model because they approximate the local feature importance of the predictive model. These approaches provide some flexibility but tend to be unstable and less spatially interpretable in high-dimensional medical images [30,31].

Unlike model-agnostic techniques, these model-specific techniques work in deep neural networks and provide pixel-level or region-based explanations. Heatmaps based on gradient-weighted class activation mapping (Grad-CAM) and attention are most commonly used to visualize salient regions. These help in the prediction of models related to brain tumor imaging. Although these approaches provide a visual and intuitive rationale, recent findings suggest that saliency maps do not always represent model reasoning truthfully and should be viewed cautiously [30,32].

To clarify the distinction between explainability techniques and their clinical applicability, Table 4 provides a comparative overview of representative XAI methods applied in the field.

The analysis in Table 4 shows that no single XAI technique can satisfy clinical requirements across transparency, reliability, and usability. Methods that are not model-specific, including LIME and SHAP, are highly flexible and can be applied across a range of models. However, they are often unstable and incur high computational costs when applied to high-dimensional medical imaging data. In contrast, model-specific methods, such as Grad-CAM and attention-based techniques, provide more intuitive spatial explanations in accordance with imaging tasks. However, they still suffer from saliency bias and dependence on the model architecture.

The comparison revealed that no single XAI method was sufficient on its own. Model-agnostic methods (LIME, SHAP) offer flexibility but suffer from instability and poor scalability for high-dimensional medical images. Model-specific methods (Grad-CAM, attention) produce intuitive spatial visualizations but are subject to saliency bias and architectural dependence. Recent studies have shown that saliency maps can appear reasonable even when generated from randomly initialized networks [30,32]. The practical consequence is that XAI outputs should be treated as hypotheses to be verified, not as confirmations of model correctness. Selecting an XAI method is a methodological design choice within the development pipeline and not a substitute for the validation steps discussed in Section 6.3.

### 6.3. Clinical Trust, Validation, and Limitations of XAI

The most important conceptual point in this section is that XAI is not evidence of clinical validity. A model that produces visually compelling saliency maps or coherent SHAP attributions can still be incorrect, biased, miscalibrated, or unsafe for deployment. Recent studies have shown that attractive explanations can actively foster false confidence in clinicians when not paired with rigorous independent evaluations [33]. Saliency maps generated from randomly initialized networks have been shown to look qualitatively similar to those from trained networks; SHAP values are unstable under input perturbations; and attention weights do not necessarily reflect the importance of causal features. Therefore, the appearance of an explanation should not be mistaken for a guarantee that the model is reasoning correctly.

**Figure 6:**
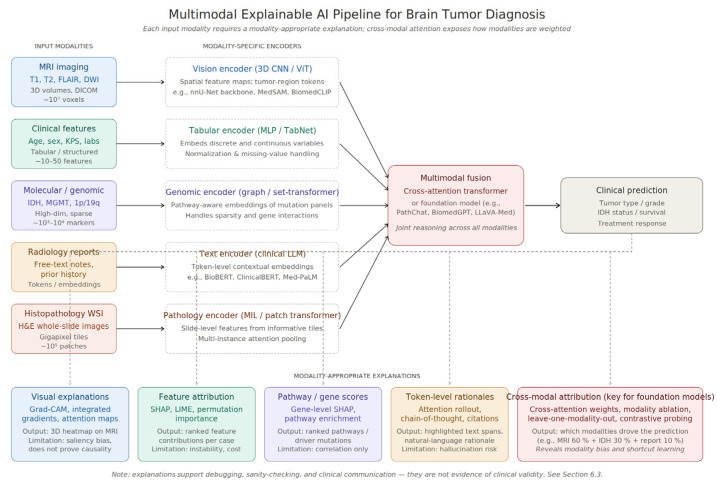
Multimodal Explainable AI pipeline for brain tumor diagnosis. Five input modalities (MRI imaging, structured clinical features, molecular/genomic markers, free-text radiology reports, and histopathology whole-slide images) were processed using modality-specific encoders, fused via a cross-attention transformer or multimodal foundation model (e.g., BiomedGPT, LLaVA-Med, PathChat), and routed to a clinical prediction head. Each modality requires its own explanation method (lower row): visual-saliency maps for MRI, SHAP-style feature attribution for tabular data, pathway/gene scores for genomics, token-level rationales for text, and slide-level attention for pathology. Cross-modal attribution (right) is the explanation type unique to multimodal systems: it identifies which modalities drive a given prediction (e.g., MRI 60% + IDH 30% + report 10%) and exposes modality bias and shortcut learning. As emphasized in Section 6.3, these explanations support debugging, sanity-checking, and clinical communication but are not, by themselves, evidence of clinical validity.

Establishing clinical validity requires evidence that XAI alone cannot provide. Specifically, XAI does not replace any of the following: (i) external validation on independent multi-institutional cohorts, including out-of-distribution scanners, vendors, and patient demographics; (ii) calibration assessment, which evaluates whether predicted probabilities match observed outcome frequencies; (iii) uncertainty estimation, which gives the model a principled way to flag low-confidence cases for human review; (iv) prospective evaluation in the actual clinical workflow, including reader studies and silent-mode deployments; and (v) clinical-utility studies that measure whether AI-assisted decisions improve patient-relevant endpoints such as time-to-diagnosis, diagnostic concordance, treatment selection, or survival. Currently, there is no standardized evaluation metric for explanation quality; published proposals for faithfulness, stability, and clinical plausibility have not converged into a single accepted benchmark. Until they do, the most defensible practice is to use XAI to support development and post hoc auditing while relying on the validation pathways above to establish whether a model is fit for clinical use. Because brain tumor diagnosis increasingly relies on multimodal evidence (MRI, clinical history, molecular markers, radiology reports, and histopathology) XAI in this setting cannot be reduced to image heatmaps alone. Figure 6 presents a multimodal XAI pipeline that pairs each input modality with an appropriate explanation method and adds cross-modal attribution as the explanation type specific to multimodal foundation models.

Reading Figure 6 left to right shows the data flow: each modality is embedded by a modality-specific encoder (3D CNN/ViT for MRI, tabular encoder for structured features, set/graph transformer for genomics, clinical LLM for text, and multi-instance learning for histopathology), and the embeddings are jointly reasoned over by a multimodal fusion module that may itself be a foundation model. Reading the bottom row shows the explanation strategies, each tailored to the data type: spatial heatmaps are useful for images but not for genomic markers, where pathway-level attribution is more interpretable; SHAP and LIME suit tabular features but generate noisy outputs on high-dimensional images; and LLM-generated rationales for the report modality must be checked against hallucination. Crucially, the cross-modal attribution box on the right distinguishes multimodal XAI from classical image-only XAI: it makes visible which modalities the model actually used, which is essential for detecting shortcut learning (for example, a model relying on report keywords rather than imaging) and modality bias.

### 6.4. Summary and Link to Generative AI

In summary, XAI is a useful but limited tool for developing brain tumor AI systems. Existing methods (LIME, SHAP, Grad-CAM, attention rollout, and cross-modal attribution) provide partial views of model behavior and can support debugging, dataset auditing, and clinical communication. However, they do not, on their own, establish that a model is robust, well calibrated, fair, or clinically useful. Clinical translation requires the combination of XAI with external validation, calibration, uncertainty quantification, prospective evaluation, and clinical utility studies. The next section addresses a separate but related challenge involving data scarcity and dataset bias, which are addressed by generative AI techniques.

## 7. Generative Artificial Intelligence for Brain Tumor Imaging

### 7.1. Motivation for Generative AI in Brain Tumor Imaging

Conventional data augmentation methods like rotating, scaling, and changing intensities add limited variation and do not introduce new anatomical or pathological patterns. In contrast, generative models strive to create medical images that maintain structural and other consistency which enrich the training set distribution. As shown by recent studies, high-quality synthetic images can improve the generalization of some models, especially for rare tumor subtype and imbalanced data cases [34,35]. As such, generative AI is seen as a powerful enabler of strong and clinically reliable brain tumor analytical pipelines.

### 7.2. GAN-Based Image Generation: Achievements and Limitations

Early generative models in medical imaging were primarily based on Generative Adversarial Networks (GANs). Data augmentation obtained from conditional GANs and StyleGAN-based architectures has been found to be effective for generating brain MRI scans and tumor subregions [35].

However, GAN-based approaches have limitations in terms of training stability, mode collapse, and difficulties in retaining fine-grained anatomical details. These problems are particularly relevant to medical imaging, where subtle structural errors can produce clinically misleading artifacts [36,37]. Thus, GAN-generated images are unlikely to be reliable for safety-critical neuro-oncological applications.

### 7.3. Diffusion Models for High-Fidelity Medical Image Generation

Recent advances have positioned diffusion models as state-of-the-art alternatives to GANs for medical image synthesis. Unlike adversarial training, diffusion-based models produce images through a progressive denoising process. This new technique has been shown to produce higher visual fidelity, training stability, and anatomical consistency [36,38].

In terms of brain tumor imaging, diffusion models have shown strong capabilities in generating realistic MRI scans that minimally store tumor morphology and spatial coherence with few artifacts [34,38]. Owing to these properties, diffusion models are suitable for data augmentation, domain adaptation, and cross-institutional generalization in neuro-oncology.

To highlight the practical differences between generative paradigms in medical imaging, Table 5 provides a comparative analysis of GAN-based approaches and diffusion models across key factors relevant to clinical applications, including training stability, image quality and anatomical consistency.

Table 5 shows that diffusion models generally outperform GAN-based approaches in terms of training stability, image quality, and anatomical consistency. While GANs have played a significant role in the early development of medical image generation, their known limitations, including training instability and susceptibility to mode collapse, restrict their suitability for safety-critical clinical applications.

In contrast, diffusion models are more stable and reliable high-fidelity models that are well suited for data augmentation and robustness enhancement in neuro-oncology. In this comparison, a significant shift toward diffusion-based generative models for clinically relevant applications is observed.

**Figure 7:**
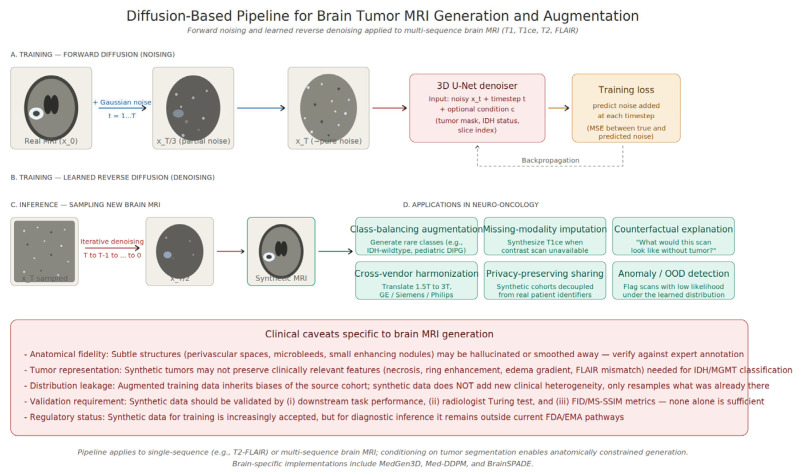
Diffusion-based pipeline for brain-tumor MRI generation and augmentation. Panel (**A**) illustrates forward diffusion: a real brain MRI is progressively corrupted by Gaussian noise over T time steps until it approximates pure noise. Panel (**B**) shows that a 3D U-Net denoiser was trained to invert this process, optionally conditioned on a tumor mask, IDH status, or slice index. Panel (**C**) illustrates the inference: starting from the sampled noise, the trained denoiser iteratively recovers a synthetic brain MRI. Panel (**D**) summarizes six brain-tumor-specific applications: class-balancing augmentation for rare entities (e.g., IDH-wildtype, pediatric DIPG), cross-vendor harmonization (1.5 T/3 T, GE/Siemens/Philips), missing-modality imputation (e.g., T1ce when contrast is unavailable), privacy-preserving cohort sharing, counterfactual explanation (tumor-free counterfactuals of a real scan), and anomaly/out-of-distribution detection. The lower panel summarizes the clinical caveats specific to brain MRI generation. The brain-specific implementations referenced in this section include MedGen3D, Med-DDPM, and BrainSPADE.

The illustrative example in the figure depicts a single client instance generated using a different imaging modality. However, the diffusion-based method is applicable to brain tumor MRI, which will aid in increasing data diversity and model generalization. Figure 7 illustrates a diffusion-based pipeline applied directly to brain tumor MRI, showing how a real preoperative scan is gradually corrupted by Gaussian noise during the forward process and then iteratively denoised by a learned U-Net to produce a synthetic but anatomically plausible MRI volume. The figure also enumerates the brain tumor-specific applications and caveats that follow from this pipeline.

### 7.4. Clinical Impact, Risks, and Open Challenges

Generative models promise a host of benefits but also entail significant clinical risks. Synthetic data can hallucinate non-existent features, reinforce the biases of the dataset, or introduce subtle artifacts that are difficult to spot visually [35,37]. Consequently, the clinical validity of brain-tumor diagnosis relying on synthetic images may be undermined in safety-critical applications.

Recent studies have highlighted the necessity for stringent validation protocols, which include expert review, statistical similarity of input and output, and evaluation of downstream clinical tasks, so that the generative model augments and does not skew learning [34,36]. To address this challenge, we discuss the integration of generative models with collaborative learning privacy-preserving strategies in the next section.

## 8. Federated Learning for Privacy-Preserving Brain Tumor Analysis

The development of strong AI models in the field of brain tumor analysis is fundamentally limited by privacy laws, ethical problems, and institutional policies regarding data sharing at a central location. Neuroimaging data are commonly distributed across different hospitals and with heterogeneous acquisition protocols and patient populations, making them unsuitable for conventional centralized training. Federated learning is a promising arrangement that allows participants from several institutions to train a model without sharing patient data but keeping it at their site. This is ideal for satisfying privacy, governance, and regulatory requirements.

### 8.1. Motivation and Privacy Challenges in Neuro-Oncology

The clinical datasets of brain tumors are sensitive and regulated by laws such as GDPR and HIPAA, preventing aggregation between institutions. Many AI models are trained on single-center or benchmark datasets, which are not always generalizable. Federated learning (FL) overcomes these limitations by requiring that model training occur locally at the participating sites, allowing only model updates to be aggregated rather than raw training data. Newer studies suggest that FL can be used practically to leverage multi-center diversity without infringing on data privacy. This is relevant to neuro-oncology applications, which require wide population coverage and robustness [10,39].

### 8.2. Federated Learning Paradigms and Applications

Federated Learning (FL) systems generally work on the paradigm of central server aggregation (e.g., FedAvg), wherein local models trained at hospitals are synchronized periodically by a coordinating server. Researchers have proposed alternative decentralized and personalized FL strategies to alleviate the effects of non-IID distributions in medical imaging. FL has demonstrated competitive performance compared to centralized training while preserving data locality in the context of segmentation and classification tasks for brain tumors. [40,41].

The table summarizes the target tasks, data distribution settings, learning strategies, and benefits/limitations to enable a structured comparison of FL methods in the MDI.

According to Table 6, federated learning demonstrates how cooperating institutions can build a model without sharing data, which respects privacy constraints in medical imaging. The study also exposes several implementation problems, such as high communication costs, increased complexity of the system, and data heterogeneity across locations.

This trade-off shows that while FL increases data accessibility and collaboration, the real-world deployment of FL requires careful system design for communication efficiency and model robustness against diverse data sources. These findings indicate that future studies should focus on optimizing communication protocols and developing strategies to cope with heterogeneity in distributed clinical settings.

### 8.3. System Architecture and Deployment Considerations

**Figure 8:**
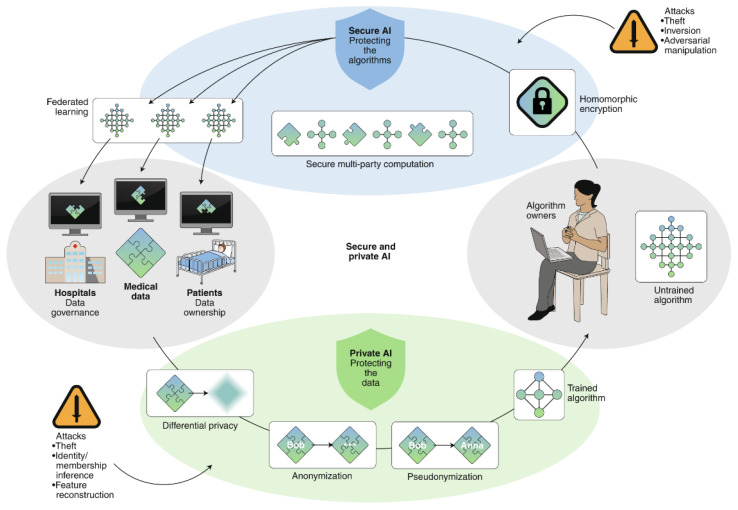
Federated learning workflow across multiple institutions.

The illustration in Figure 8 displays the workflow in which FL operates with respect to neuro-oncology by collecting data centrally and federated training in a few hospitals. Within the framework of Federated Learning, local organizations do not share player MRI data. Instead, only a model update is exchanged securely and encrypted. This architecture enables learning in different clinical settings while maintaining privacy.

Figure 8 shows the collaborative training of the model among institutions using federated learning without sharing patient data. This arrangement permits compliance with regulations while utilizing data diversity to enhance the robustness of models used in the analysis of brain tumors in clinical trials.

### 8.4. Limitations and Clinical Readiness

Though several advantages, it has certain practical drawbacks. The increase in communication overhead leads to longer training times and possibly larger deep models. In addition, the non-IID data distribution across institutions not being IID may degrade convergence and fairness. A recent study showed that personalization strategies, secure aggregation of data, and clinical validation of FL models are beneficial for ensuring reliable and fair performance across sites [42,43]. Driven by these considerations, we discuss the integration of FL with other complementary approaches, such as multimodal data fusion.

## 9. Multimodal Fusion for Precision Neuro-Oncology

Precision neuro-oncology strives to customize the diagnosis, prognosis, and treatment approaches based on each patient’s individual biology. Medical imaging is valuable for spatial and morphological data on brain tumors, although it alone cannot capture the underlying molecular heterogeneity that governs tumor behavior and treatment response. Researchers have focused on multimodal fusion to overcome this limitation. Multimodal fusion is a methodology that integrates imaging data with molecular information, including genomics, transcriptomics, and proteomics.

### 9.1. Motivation for Multimodal Learning in Brain Tumor Analysis

Brain tumors, particularly gliomas, are characterized by significant molecular heterogeneity both between and within tumors. Molecular markers, such as IDH mutation status, 1p/19q codeletion, and MGMT promoter methylation, are essential for the WHO CNS5 classification and treatment planning. Although advanced imaging provides indirect indicators of tumor aggressiveness, imaging features alone are often insufficient for an accurate molecular characterization. Studies that combine imaging with molecular profiles consistently report improved predictions over imaging-only baselines [44,45]. Therefore, the methodological question for this section is not whether multimodal AI is important in principle but which specific approaches are demonstrably moving the field forward in measurable terms (cohort size, fusion strategy, external validation, and prospective evidence). Section 9.2 addresses this question directly, as shown in Table 7.

### 9.2. Multimodal Fusion Strategies and Learning Paradigms

Multimodal fusion approaches can be broadly classified into three categories: early, intermediate, and late fusion. Early fusion refers to the concatenation of raw or handcrafted features from different modalities before training the model. It offers an intuitive implementation but has limited flexibility. Intermediate fusion employs modality-specific encoders, such as CNNs for imaging and MLPs or transformers for omics data, allowing the learning of shared representations that are effective for capturing complex cross-modal interactions. Late fusion aggregates the individual predictions made by the unimodal models. This strategy is often favored when data of different modalities are absent or are available asynchronously.

Three architectural trends define the current state-of-the-art multimodal brain-tumor analysis. First, intermediate fusion using cross-attention transformers has displaced naive feature concatenation, allowing the model to learn modality-specific weights that are conditioned on the input. Second, joint vision-language and vision-omics foundation models fine-tuned on neuro-oncology data have begun to enable zero-shot or few-shot transfer to held-out institutions, an outcome that single-modality benchmarks rarely achieve. Third, attention-based pooling provides a built-in modality-attribution signal that supports the cross-modal explanation methods discussed in Section 6.3 [48]. However, the demonstrated improvements in published multimodal studies remain modest in absolute terms compared with strong unimodal baselines, and external validation evidence is the exception rather than the rule, as qualitatively summarized below.

Table 7 addresses the question directly: which multimodal approaches are moving the field forward? It curates seven representative studies and reports, for each, the integrated modalities, fusion strategy, target clinical task, external-validation posture, and key translational limitation. This qualitative format makes it possible to read off, at a glance, the architectural family represented by each study and the gaps that remain. As explicitly stated below the table, quantitative metrics (cohort sizes, confidence intervals, performance drops) are not tabulated because they were not uniformly reported in compatible form across the cited sources; this limitation is reiterated in Section 13.1.

Several qualitative patterns emerge from Table 7 that clarify the current status of the multimodal field. First, datasets are dominated by public benchmarks (TCGA-derived cohorts and BraTS-related collections), and large prospectively curated multi-institutional multimodal cohorts remain rare. Second, the fusion strategy is converging away from naive early-stage concatenation toward intermediate cross-attention architectures and, most recently, multimodal foundation-model joint embeddings. Third, external validation is the most important divider across the table: studies that report only internal cross-validation are most exposed to the benchmark-to-clinic gap, whereas studies that include multi-cohort or held-out-site evaluation provide more credible (if still partial) generalization evidence. Fourth, prospective clinical-utility evidence remains effectively absent; none of the representative entries correspond to a published prospective trial measuring whether multimodal AI predictions changed treatment decisions or improved patient outcomes. We emphasize that Table 7 is a qualitative synthesis rather than a meta-analysis, as quantitative performance metrics across these representative studies are not uniformly reported in a compatible format. Therefore, we avoided tabulating numerical values that could not be consistently traced across all cited sources.

This comparison shows that while multimodal models provide enhanced diagnostic and prognostic power, their actual implementation is complicated by data alignment, missing modalities, and scalability issues. Such investigations imply that future research efforts must aim to develop robust data fusion strategies for incomplete heterogeneous datasets and clinically relevant outcomes.

### 9.3. Multimodal Pipelines and Clinical Applicability

Translating the approaches listed in Table 7 into clinical deployment requires solving three concrete obstacles. First, modality heterogeneity: Imaging, molecular, and clinical data differ in dimensionality, acquisition protocol, and missingness rate; therefore, the system must support modality dropout at inference time without retraining. Second, cohort scale: multimodal cohorts are typically smaller than imaging-only cohorts in the published literature, making overfitting and underpowered comparisons a significant risk. Third, interpretability: cross-modal models must expose which modality drives a given prediction, both for clinician trust and for detecting shortcut learning (e.g., a model that ignores imaging and relies entirely on a structured clinical feature). Modular architectures with attention-based fusion are the current best response to all three because they support modality dropout naturally, can be pretrained on each modality separately to mitigate scale, and provide modality-attribution signals as a by-product of the attention mechanism [49,50].

**Figure 9:**
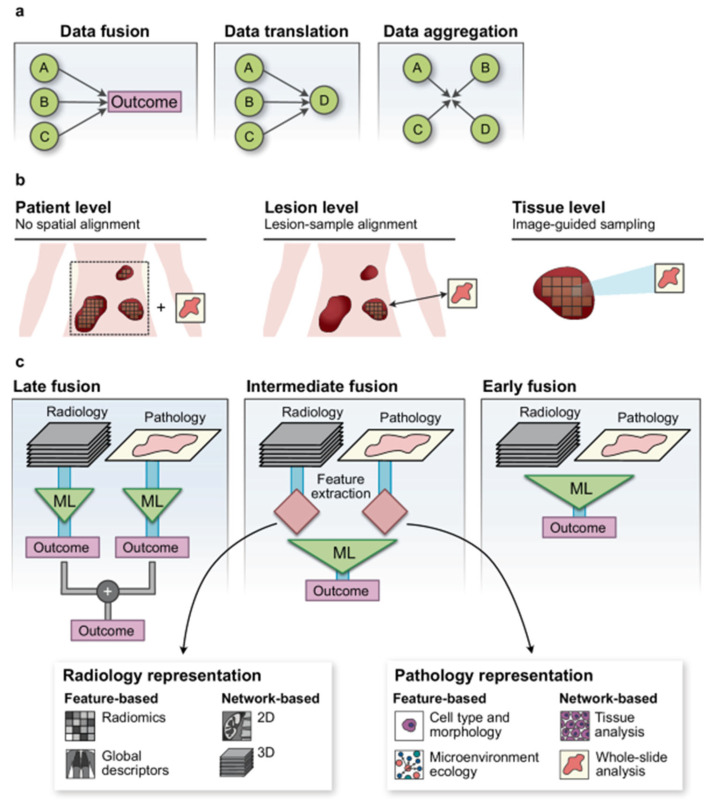
(**a**) Three multimodal data integration strategies: data fusion combines multiple input sources (A, B, C) to predict a single outcome; data translation maps inputs to an intermediate representation (D); and data aggregation merges multiple sources (A, B, C, D) without a designated output node. (**b**) Spatial alignment levels for integrating radiology and pathology data: patient level (no spatial alignment), lesion level (lesion-sample alignment), and tissue level (image-guided sampling). (**c**) Multimodal fusion pipeline for precision neuro-oncology. Imaging features extracted by a vision encoder are combined with molecular, clinical, or text representations through a fusion module—early concatenation, intermediate cross-attention, or late ensemble—producing a joint representation that drives clinical predictions such as tumor type, molecular status, or prognosis. Specific instantiations of this pattern are listed in Table 7, along with their cohorts, metrics, and validation statuses.

Figure 9 illustrates the architectural patterns shared by the studies in Table 7. It shows where each modality enters the pipeline and the alternative stages at which fusion can be performed (early, intermediate, or late), so that the reader can map a specific row of Table 7 onto the corresponding fusion stage in the figure.

To read Figure 9 against Table 7, studies using simple feature concatenation [46] correspond to the early fusion path, intermediate cross-attention designs [44,47,48] correspond to the central fusion block with modality-specific encoders, and foundation-model joint embeddings [50] correspond to a single multimodal encoder that subsumes both modality-specific encoding and fusion. The figure thus serves as a navigation aid, not a new architectural proposal; it makes the fusion choices in Table 7 visible in a single diagram.

### 9.4. Challenges and Future Directions

The gaps identified in Table 7 define the immediate research agenda for multimodal brain-tumor AI. First, cohort size: large, prospectively curated multi-institutional cohorts that pair imaging with omics and clinical outcomes are still rare. Federated learning across consortia (Section 8) is the most realistic path for assembling them without compromising privacy. Second, external validation: Only a subset of recent studies report leave-one-site-out or zero-shot transfer; this should become a publication standard rather than an exception. Third, missing modalities: most cohorts are incomplete (e.g., not every patient has RNA-seq), and the field needs principled handling beyond ad hoc imputation, including modality dropout training and explicit uncertainty quantification when a modality is absent. Fourth, prospective clinical-utility studies: not a single entry in Table 7 corresponds to a published prospective trial measuring whether multimodal predictions changed treatment decisions or improved patient outcomes; this is the single most important missing piece of evidence. Fifth, evaluation frameworks: Standard reporting that jointly considers discrimination, calibration, fairness across subgroups, and clinical decision impact should replace the current accuracy-centric reporting. Recent studies advocate the combination of multimodal fusion with federated learning and XAI to produce trustworthy and scalable precision neuro-oncology systems [51,52]. Addressing these five gaps would make such systems credible for clinical deployment.

## 10. Computational Efficiency and Sustainability (Green AI)

To enhance performance, the complexity of the proposed deep learning schemes for brain-tumor deciphering is continuously increasing. Consequently, they monopolize GPU resources, have lengthy training processes, and consume high power requirements. Although these models tend to deliver the best results on benchmark datasets, their operational and environmental costs raise serious concerns about scalability, sustainability, and clinical deployment. In this regard, Green AI has emerged, which tackles these challenges with a focus on energy-efficient, resource-aware, and practically deployable AI.

Recent research on performance improvements using more complex architectures suggests that these gains come at the cost of significantly greater computation. A small improvement in accuracy, for example, may necessitate a significant increase in training time and power. Because the clinical setting where medical imaging AI must be accurate, fast, stable, and cost-effective, this trade-off is important in this context. Heavy computational needs could hinder real-time inference or deployment in resource-restricted hospitals, limiting large-scale clinical uptake [11,53].

From a system perspective, we observe inefficiencies at various stages in the AI pipeline, starting from data pre-processing to model training to hyperparameter tuning to inference. The iterative computation and memory demands of large transformer-based and diffusion-inspired architectures exacerbate these challenges. Consequently, recent studies and research works have focused on the efficiency of the model. Techniques such as model compression, model pruning, quantization, lightweight architectures, and early exit strategies have been studied. These strategies help reduce the computational burden without a major drop in predictive performance [54,55].

Moreover, AI systems and algorithms must have minimal energy consumption and carbon footprints, along with computational efficiency. Advocates of Green AI argue that, in addition to accuracy reporting, an indicator of resource usage should also be declared. These include the training time, energy cost, and type of hardware used [56]. The aforementioned change in evaluation philosophy fits well with the medical AI deployment requirements for sustainability, cost transparency, and long-term maintenance [11].

The growing trend towards multistage pipelines with segmentation, classification, explainability, and multimodal fusion necessitates a delicate balance between performance and efficiency for brain tumor analysis. Without efficient design, these pipelines can become impractical for routine clinical applications. Future AI systems in neuro-oncology must be designed with computational constraints in mind as a first-class objective and not as a perfunctory secondary optimization step.

## 11. From Benchmark Datasets to Real-World Clinical Data

Benchmark datasets have played a central role in advancing brain tumor analysis by providing standardized datasets, annotations, and evaluation protocols. Datasets such as BraTS have facilitated a fair comparison of algorithms and have resulted in rapid innovations in methodology. Accumulating evidence suggests that strong performance on curated benchmarks does not guarantee robust or clinically reliable behavior of models when deployed in real-world healthcare settings.

A major limitation of benchmark datasets is that they are collected under controlled conditions with uniform annotations and poor clinical diversity. Imaging protocols, scanner vendors, reconstruction pipelines, and patient demographics are often curated or implicitly normalized in benchmarks and are not reflective of heterogeneity in clinical data. Thus, models that are trained and validated only on benchmark datasets appear to overestimate their generalization performance and do not perform well under domain shifts [19,20]. Table 8 contrasts the benchmark datasets with real-world clinical data across the key evaluation aspects.

Table 8 contrasts benchmark and real-world settings along seven aspects, with each row backed by representative empirical studies that characterized the gap. Benchmark datasets, most prominently the BraTS challenge series, have been instrumental in advancing brain tumor algorithm development by providing standardized, well-annotated cohorts for fair comparison [6,28]. However, the same standardization that makes benchmarks useful for ranking systems is what limits their fidelity to clinical practice: clinical data are acquired across heterogeneous scanners and protocols [17,18], include incomplete modalities [3,15], and reflect a broader patient population than the typical benchmark cohort [20,63].

The second concern is label reliability. Benchmark datasets are typically annotated using expert consensus protocols that yield high inter-rater agreement at the cost of compressing genuine diagnostic uncertainty into a single ground-truth label [21,28]. Real-world clinical data are messier: ground truth often emerges incrementally as molecular profiling, surgical pathology, and follow-up imaging accumulate, leading to delayed and partially missing outcome labels. Models trained on curated benchmark labels can therefore encounter forms of label noise and label shift to which they were never exposed during training, complicating calibration and uncertainty estimation in clinical use [64].

The third point in Table 8, overestimated generalization, is supported by direct empirical evidence. Multi-institution evaluations of brain tumor segmentation report substantial cross-site performance drops [17], and cross-hospital evaluations of deep learning imaging models have documented similar degradation [19]. Across the surveyed literature, formal external validation and prospective evaluation remain the exception rather than the rule. Closing this gap requires multicenter cohorts, prospective evaluation, and clinically relevant endpoints beyond accuracy [3,27,63].

Recent studies have focused on closing the gap between benchmark-driven research and clinical deployment. The shared recommendations across these studies are external validation on independent cohorts, evaluation under controlled domain shifts, transparent reporting of failure modes alongside successes, and closer collaboration between AI researchers and clinicians from study design to post-deployment monitoring [3,19,27,63].

## 12. Open Research Challenges and Future Perspectives

Although technology for using AI to analyze brain tumors is advancing, clinical translation is limited by many research issues. Due to heterogeneous clinical environments, previously unseen acquisition protocols, and patients from various demographic backgrounds, models usually suffer performance drop-off at later stages. Thus, the major remaining challenges are robustness and generalizability. By shifting from benchmarks to robustness-aware validation, which models domain shifts and clinical variability, we can overcome this limitation.

Another significant challenge is the availability and reliability of clinically representative datasets. Generative models and federated learning systems address the concerns of data scarcity and privacy. However, they also introduce risk problems. These systems create problems of fidelity of the generated data, amplification of bias, and added complexity of the system. Whether these approaches distort disease characteristics while preserving clinically meaningful patterns remains an open research problem.

Table 9 summarizes the key limitations of the survey, together with future perspectives. Some open challenges and possible future research directions on AI-based brain tumor research are presented in Table 9.

Table 9 discusses the future directions for brain tumor analysis in the age of AI to become a reality in the clinic. The aforementioned challenges necessitate research frameworks that are holistic in nature, emphasize the improvement of robustness, trustworthiness, and computational and clinical feasibility, and are conducted as joint studies rather than in isolation.

Indicative threshold values can help define performance targets for clinical translation. Because of the practical implications of longer inference times and standard interactive workflows, inference times below one second per scan are usually considered appropriate for everyday clinical workflows. Model robustness is usually confirmed by its continued performance across datasets from different institutions or protocols. While there is currently no gold standard, a drop of less than 5–10% under domain shift is often considered acceptable for clinically reliable deployment.

In addition to performance, interpretability and trust are important adoption factors. The future of XAI must go beyond post hoc visualization approaches and offer verifiable and stable orientations in line with clinical reasoning. Uncertainty quantification, causal modeling, and clinician involvement can improve trust in the use of AI in healthcare.

From a system-level perspective, as AI pipelines become more complex owing to multimodal fusion, generative augmentation, and privacy-preserving learning, computational efficiency and sustainability are becoming increasingly critical. Integrate efficiency-aware design principles to support scalable deployment across diverse healthcare settings.

The advancement of precision neurology relies on collaborative research and evaluation across large international hospital networks. The development of this field calls for integrated evaluation frameworks that evaluate predictive performance, robustness, interpretability, efficiency, and clinical impact. Collaboration between AI researchers and clinical and regulatory communities will power safe and effective clinical AI systems.

Moreover, it is important to highlight the practical implications of this survey for AI and clinical practitioners, in addition to the previously identified research challenges.

### Practical Implications for AI and Clinical Practitioners

This survey will be useful for AI researchers and clinicians. The suggested classification scheme was devised to help people associated with the development of AI models understand how the diagnostic task, learning paradigm, and data modality interact with one another. To build a clinically reliable and generalizable AI system, domain shift and robustness, as well as dataset bias, are important challenges that must be addressed.

In addition, this survey identified several concrete implications for clinical workflow integration. For radiologists and neuro-oncologists, the structured comparison of benchmark and real-world conditions (Section 5 and Section 11) clarifies which AI claims should be regarded as preliminary and which carry stronger external validation evidence. For AI developers, the recurring shortfalls highlighted in Section 5, Section 9 and Section 11—limited external validation, absent prospective evaluation, and insufficient scanner and vendor diversity—define the most actionable directions for closing the gap between published performance and deployable systems.

Essentially, this article details the development process of the AI system and its clinical use, bridging the technical and clinical aspects of precision neuro-oncology.

## 13. Limitations of the Study

The recommendations of this survey should be considered in light of several limitations. We discuss these in five subsections so that the reader can assess what this study does and does not establish.

### 13.1. Narrative Review Scope and Bibliometric Depth

This work is presented as a structured narrative review with a lightweight bibliometric overview (Section Bibliometric Overview of the Reviewed Literature, Table 1, Figure 3) rather than as a full scientometric study or formal systematic review. We searched peer-reviewed journal articles and full conference proceedings indexed in IEEE Xplore, PubMed, Scopus, and Google Scholar between January 2018 and June 2025. The proportions reported in Section Bibliometric Overview of the Reviewed Literature are approximate and intended to provide qualitative context for the surveyed literature; they do not constitute meta-analytic estimates. A full PRISMA-compliant screening with multiple independent reviewers, formal inter-rater agreement, scientometric or network analysis (e.g., VOSviewer-style citation mapping), and supplementary material listing each included study by category was not performed and would require a dedicated systematic review effort. We acknowledge this as a methodological limitation and recommend a dedicated systematic review with a formal bibliometric analysis as valuable future work. The qualitative observations presented throughout this review (e.g., that external validation is the exception rather than the rule, prospective evaluation is rare, and adult diffuse gliomas dominate the AI literature) are widely supported by the cited primary studies and adjacent surveys but should be interpreted as patterns observed in the surveyed literature rather than as outputs of a formal meta-analytic procedure. Heterogeneous reporting in the primary literature (absent confidence intervals, undisclosed scanner metadata, and varying outcome definitions) further constrains formal statistical pooling. New methods and datasets continue to appear at a rapid pace, and approaches published after June 2025 are not included.

### 13.2. Limited Engagement with Formal Clinical Guidelines

The 2021 WHO Classification of Tumors of the Central Nervous System (CNS5) reorganized brain tumor diagnosis around integrated histological-molecular categories (Section 2), and the Response Assessment in Neuro-Oncology (RANO) criteria were updated to RANO 2.0 in 2023, with a parallel AI-RANO initiative seeking to standardize AI-assisted response assessment. The studies surveyed in Section 4, Section 5, Section 6, Section 7, Section 8 and Section 9 implicitly used the WHO CNS5 framing when predicting IDH, MGMT, or 1p/19q status, but rarely reported whether their evaluation cohorts and outcome definitions conformed to current RANO 2.0 or AI-RANO conventions. This review tabulates AI methods at the algorithmic level; a deeper guideline-aligned analysis—classifying every reviewed study by its compliance with the latest WHO CNS5 categories, RANO 2.0 measurable disease and pseudo-progression criteria, and AI-RANO reporting recommendations—was not performed and would be a useful follow-up review.

### 13.3. Limited Coverage of Regulatory Pathways

Clinical-grade deployment of AI in neuro-oncology requires clearance under specific regulatory regimes—FDA 510(k) or De Novo in the United States, CE marking under MDR in the European Union, and equivalent national pathways in other jurisdictions—together with conformance to standards such as Good Machine Learning Practice and the FDA-recognized predetermined change control plans for adaptive AI. This survey does not enumerate which of the reviewed methods have advanced through these pathways, what locked-versus-adaptive deployment posture they declare, or what post-market surveillance plans they specify. As of mid-2025 the number of FDA-cleared neuro-oncology AI devices remains small relative to the volume of published methods, and a structured registry-based review of cleared products is outside the scope of this manuscript.

### 13.4. Limited Engagement with Real-World Clinical Adoption Evidence

The qualitative robustness assessment (Section 5 and Section 11) exclusively draws on published technical reports. Evidence about actual adoption—integration into PACS workflows, reader-study evidence comparing AI-assisted and standard-of-care reads, time-to-diagnosis and treatment-decision impact, post-market surveillance data, and equity audits across patient subgroups—was not systematically reviewed here because the published peer-reviewed literature on these endpoints in neuro-oncology AI is currently sparse. The qualitative observation that prospective evaluation is the exception across the surveyed literature is itself part of the explanation: the evidence is simply not yet available at the volume required for a structured clinical-readiness review.

### 13.5. Implications for the Scope of the Present Review

Section 13.2, Section 13.3 and Section 13.4 indicate that this manuscript does not constitute a full critical review of clinical readiness in the strict sense used in the clinical translation literature. What it establishes, and what we believe is the manuscript’s defensible contribution, is a comprehensive technical review of AI methods for brain tumor imaging, structured by a three-factor taxonomy and grounded in transparent bibliometric and qualitative-robustness reporting. The accompanying clinical framing in Section 2 and the deployment-gap discussion in Section 5 and Section 11 are intended to position this technical review against clinical reality, not to substitute for the dedicated clinical, regulatory, and adoption reviews that the field still requires. Accordingly, we avoided framing the title or abstract as a critical clinical-readiness assessment; the contribution is more accurately described as a comprehensive technical and methodological survey with explicit acknowledgement that several adjacent reviews remain to be written.

## 14. Conclusions

This survey provides a comprehensive technical review of the artificial intelligence approaches for brain tumor analysis. A three-factor taxonomy organizes the literature by diagnostic task, learning paradigm, and data modality. This work is presented as a structured narrative review with an explicit acknowledgement of its scope (Section 13.1).

The evaluation lens of this survey extends beyond accuracy to robustness, generalization, and barriers to clinical adoption. The qualitative robustness summary in Table 3 highlights that external validation is reported only sporadically, benchmark dependence is pervasive, and scanner and vendor diversity is insufficient in most representative studies. Across the surveyed literature, formal external validation and prospective evaluation remain the exception rather than the rule. Explainable AI is positioned as a debugging and communication tool, not as evidence of clinical validity (Section 6). Generative models, especially diffusion-based models, are reviewed as a means to address data scarcity rather than as substitutes for real clinical heterogeneity (Section 7).

Multimodal fusion (Section 9) is identified as the most consequential frontier. Cross-attention transformers and emerging foundation models pair imaging with molecular, clinical, and text modalities; however, reported gains over strong unimodal baselines remain modest, external validation is the exception, and prospective clinical-utility evidence is effectively absent. Computational efficiency and sustainability (Section 10) are becoming increasingly relevant as pipelines grow.

Taken together, the survey demonstrates that any responsible appraisal of brain tumor AI must consider predictive performance, robustness, calibration, interpretability, computational cost, and clinical feasibility. As acknowledged in Section 13, this manuscript is a comprehensive technical and methodological review rather than a full critical assessment of clinical readiness; dedicated regulatory, guideline-aligned (WHO CNS5, RANO 2.0, AI-RANO), and adoption-evidence reviews remain to be written and would complement this work. With these scope qualifications, the survey is intended as a structured reference for researchers and clinicians working toward the safe and effective translation of AI into neuro-oncology practice.

Looking ahead, we anticipate a shift away from standalone narrow AI systems toward clinically integrated multimodal decision-support systems that leverage continuous learning under predetermined change-control plans. Federated learning, generative modeling, and explainable AI, alongside guideline-aligned evaluation protocols (WHO CNS5, RANO 2.0, AI-RANO) and prospective clinical-utility studies, define the path from benchmark performance to clinical translation.

## Figures and Tables

**Table 1:** Approximate bibliometric distribution of the reviewed literature on AI for brain tumor analysis, with key observations for each category.

Category	Approximate Proportion	Key Observation
Tumor detection/segmentation	Approximately one third	The largest single category was dominated by BraTS-derived cohorts and U-Net family architectures.
Classification (tumor type or grade)	Approximately one quarter	Most CNN-based methods are reliant on public datasets and have limited scanner diversity.
Radiogenomics/molecular prediction	A meaningful minority	IDH, MGMT, and 1p/19q prediction predominated, with cohort sizes typically in the low hundreds.
Survival prediction and prognostic modeling	A smaller minority	Often based on TCGA-LGG/GBM; few studies have reported external validation cohorts.
Treatment-response assessment	A limited proportion	Underrepresented despite high clinical relevance; mostly retrospective post hoc analyses.
Post-treatment MRI monitoring	Very limited representation	An emerging focus, supported by recent benchmarks such as BraTS 2024 post-treatment glioma.
Pediatric brain tumors	A small minority	Recent attention has been paid to BraTS-PEDs; pediatric gliomas are biologically distinct and are not miniature adult tumors.
Brain metastases	A small minority	Smaller, more heterogeneous cohorts than glioma studies; limited multicenter evaluation.
Meningioma studies	Very limited representation	Markedly underrepresented relative to its clinical incidence.
Studies using BraTS or other public benchmarks	The majority	This reflects a strong benchmark dependency that may overstate clinical generalization.
Single-center or single-dataset evaluation	The majority	Limitations include the assessment of scanner, vendor, and demographic generalization.
Studies reporting external validation	A limited proportion	External validation remains an exception rather than the rule.
Prospective evaluation in a clinical workflow	Very rare	Most of the reviewed studies were retrospective, and prospective deployment evidence was largely absent.

**Table 2:** Mapping representative AI studies within the proposed taxonomy.

Ref.	Diagnostic Task	Methodology	Data Type	Main Contribution	Key Limitation
[2]	Feature analysis	ML + Radiomics	MRI	Quantitative, interpretable imaging features	Single-center radiomics features; lacks external validation and scanner-protocol diversity
[3]	Prognosis, treatment	Multimodal AI	Imaging + Clinical	Precision neuro-oncology overview	Conceptual review without quantitative pooled benchmarks or prospective deployment evidence
[6]	Detection	CNN	MRI	High detection accuracy	Trained/evaluated on curated public dataset only; no external cohort; no calibration or uncertainty assessment
[5]	Survey (multiple)	ML/DL	MRI	Task and method dominance analysis	Aggregates accuracy/Dice without robustness, clinical-utility, or external-validation analysis
[7]	Multiple tasks	DL (survey)	MRI	Clinical challenges overview	Narrative survey without standardized data extraction, pooled metrics, or risk-of-bias assessment
[8]	Classification	AI + Molecular	Imaging + Genomics	Molecular glioma classification	Requires specialized intraoperative SRH hardware; not transferable to standard MRI; limited multi-center generalization
[19]	Evaluation	—	Neuroimaging	Benchmark bias identification	Empirical case study of generalization failure; does not propose corrective methodology or external benchmark
[20]	Evaluation	—	Medical imaging	Translational benchmarking critique	Conceptual taxonomy of bias types without quantitative meta-analysis or empirical mitigation benchmarks
[9]	Interpretability	XAI	Model-agnostic	Trust and explainability	General XAI survey with limited brain-tumor-specific evaluation; no clinician validation studies referenced
[10]	Privacy	Federated learning	Multi-institutional	Privacy-preserving AI	High communication overhead; non-IID data degrades convergence; lacks prospective clinical FL deployment evidence
[11]	Efficiency	Green AI	Computational	Energy-aware AI design	Generic computational-efficiency framework; not validated in medical imaging or neuro-oncology pipelines

**Table 3:** Qualitative robustness and external validation characteristics of representative AI brain tumor studies.

Ref.	Clinical Task	Dataset Type	External Validation Status	Reported Generalization Issue	Key Translational Limitation
[6]	Brain tumor classification (multi-class)	Public benchmark dataset (single source)	No external validation performed	Cross-scanner robustness not evaluated; single-source benchmark used for both training and testing	Single-center cohort with limited vendor diversity
[7]	Tumor classification and detection (narrative review)	Mixed public benchmarks reviewed across cited studies	External validation only sporadically reported across reviewed studies	Performance degradation reported under external validation in reviewed evidence	Benchmark dependency limits clinical-translation evidence
[8]	Intraoperative diffuse glioma molecular classification	Stimulated Raman histology (intraoperative imaging)	External validation not performed	Cross-institution generalization not established beyond the training cohort	Requires specialized intraoperative hardware; limited transferability to standard MRI workflows
[19]	Detection of pneumonia in chest radiographs (cross-domain reference)	Multi-hospital chest radiograph cohorts	External validation performed (cross-hospital)	Performance degradation reported under external validation; model exploits site-specific signals	Empirical demonstration of cross-site generalization failure; broadly applicable to neuro-oncology AI
[20]	Bias and equity assessment across medical-imaging AI	Conceptual taxonomy across the AI medical-imaging literature	Not assessed in any single study; review highlights gaps across literature	Protocol heterogeneity sensitivity reported as a recurring source of model bias	Insufficient scanner/vendor diversity recognized as a translational barrier
[25]	Detection/classification of brain tumors (representative pipeline)	Single-center hospital cohort	External validation not performed	Cross-scanner robustness not evaluated; field-strength differences not assessed	Single-center cohort with limited vendor diversity; prospective deployment not assessed

**Table 4:** Comparison of representative XAI techniques in medical imaging.

XAI Method	Model Dependency	Explanation Output	Clinical Usefulness	Key Limitation
LIME	Model-agnostic	Superpixel importance	Local interpretability	Instability
SHAP	Model-agnostic	Feature attribution	Global + local insight	High computational cost
Grad-CAM	CNN-specific	Saliency heatmaps	Tumor localization	Saliency bias
Attention maps	Model-specific	Learned attention regions	Model transparency	Architecture dependent

**Table 5:** Comparison between GAN-based and diffusion-based generative models in medical imaging.

Aspect	GAN-Based Models	Diffusion Models
Training stability	Often unstable	Highly stable
Mode collapse	Common	Absent
Image fidelity	Moderate	High
Anatomical consistency	Limited	Strong
Suitability for medical imaging	Restricted	Highly suitable

**Table 6:** Representative federated learning studies for brain-tumor imaging.

Ref.	Task	Data Distribution	FL Strategy	Main Benefit	Key Limitation
[10]	Segmentation	Multi-hospital MRI	FedAvg	Privacy preservation	Communication cost
[39]	Classification	Cross-center MRI	Personalized FL	Improved generalization	Model heterogeneity
[40]	Segmentation	Non-IID datasets	Decentralized FL	Robustness to bias	Training complexity
[41]	Multi-task	Multi-modal centers	Hybrid FL	Scalability	Limited clinical validation

**Table 7:** Qualitative synthesis of representative multimodal fusion approaches in precision neuro-oncology.

Ref.	Modalities Integrated	Fusion Strategy	Target Task	External-Validation Posture	Translational Limitation
[44]	MRI imaging combined with omics data (transcriptomic/genomic features)	Intermediate fusion using attention-based integration of modality-specific embeddings	Glioma molecular subtype prediction	Internal cross-validation only; external multi-institutional validation not performed	Benchmark dependency; limited cohort heterogeneity
[45]	MRI imaging combined with structured clinical and demographic variables	Late fusion (decision-level ensemble of modality-specific predictions)	Overall survival prognostic modeling	Held-out internal test cohort; cross-institution evaluation not reported	Single-cohort design; prospective evaluation not assessed
[46]	MRI imaging combined with radiomic and molecular features (early-stage radiogenomics)	Early fusion (concatenation of hand-crafted and learned features)	IDH mutation status prediction	External validation not performed	Small cohort size; feature stability under acquisition shift not assessed
[47]	MRI imaging combined with histopathology whole-slide images	Cross-modal attention-based deep fusion	Glioma grading and molecular characterization (multi-task)	External validation performed across at least two public cohorts	Acquisition heterogeneity across cohorts only partially controlled
[48]	MRI imaging combined with clinical variables and multi-omics data (multi-modal integration)	Hybrid architecture with modality-specific encoders and joint fusion module	Treatment response and recurrence-free outcome prediction	Multi-center internal evaluation reported; independent prospective validation not assessed	Cohort composition skewed toward research-grade data; clinical-workflow integration not evaluated
[49]	MRI imaging combined with radiology text reports (vision–language framework)	Joint vision–language transformer encoder	Image–report alignment and downstream tumor classification	External validation across institutional report styles not performed	Text-report style and terminology variability across institutions is a recognized generalization challenge
[50]	MRI imaging combined with molecular data within a multimodal foundation-model framework	Joint embedding via large multimodal foundation model with task-specific fine-tuning	Molecular prediction (e.g., IDH/MGMT) with transfer to held-out data	Transfer evaluation to a held-out cohort reported; full prospective deployment not assessed	Foundation-model approaches require substantial compute; clinical workflow and regulatory pathways still unaddressed

**Table 8:** Benchmark datasets versus real-world clinical data in brain-tumor analysis.

Aspect	Benchmark Datasets (e.g., BraTS)	Real-World Clinical Data	Supporting Evidence
**Data acquisition**	Single or few institutions; uniform vendor and protocol within a release	Mixed scanner vendors (GE, Siemens, Philips), 1.5 T and 3 T, varying sequence parameters across sites	Bakas et al., BraTS challenge series (single-vendor protocol harmonization) [57]; Mårtensson et al., 2020 (clinical MRI heterogeneity in deep learning) [58]
**Annotation**	Expert consensus labels with iterative refinement; central QC	Inter-rater variability; evolving labels as molecular and clinical follow-up data become available; partial annotations common	Menze et al., 2015 (BraTS annotation protocol and inter-rater agreement) [59]; Visser et al., 2019 (inter-observer variability in glioma segmentation) [60]
**Patient diversity**	Skewed toward adult HGG/GBM; under-representation of pediatric, rare entities, and non-Western cohorts	Broad demographic, age, and ethnic spectrum; rare entities present at clinical base rates	Calabrese et al., UCSF-PDGM (multi-demographic glioma cohort) [15]; Koçak et al., 2025 (bias and equity in AI medical imaging) [20]
**Imaging protocols**	Resampled to a fixed resolution and skull-stripped during dataset preparation	Variable slice thickness, reconstruction kernels, motion artifacts; routine clinical preprocessing only	Kondrateva et al., 2024 (effect of preprocessing variability on glioma segmentation) [17]; Kazerooni et al., 2024 (BraTS 2023 challenge: protocol heterogeneity) [13]
**Missing data**	All four modalities (T1, T1ce, T2, FLAIR) typically present; missing scans excluded at release	Frequent absence of one or more modalities (e.g., no T1ce when contrast contraindicated); incomplete molecular profiling	Khalighi et al., 2024 (multimodal review notes incomplete data in clinical practice) [3]; Havaei et al., 2016 (HeMIS: hetero-modal imputation in clinical data) [61]
**Evaluation focus**	Dice, Hausdorff distance, accuracy, AUC on held-out splits; leaderboard ranking	Diagnostic concordance with the radiologist of record, time-to-diagnosis, treatment-decision impact, post-deployment monitoring	Park & Han, 2018 (methodologic standards for AI imaging evaluation) [62]; Wagner et al., 2024 (clinical evaluation of AI in neuroradiology) [7]
**Generalization**	Often overestimated when reported only on the same-distribution test split	Performance degradation reported under cross-scanner, cross-vendor, and cross-institution evaluation in multiple studies (see Table 3)	Zech et al., 2018 (variable generalization of pneumonia model across hospitals) [19]; Matta et al., 2024 (model generalization in medical imaging) [26]

**Table 9:** Open challenges and future research directions in AI-based brain-tumor analysis.

Research Aspect	Current Limitation	Future Research Direction
Robustness	Poor generalization under domain shift	Domain-aware and stress-tested evaluation
Data availability	Limited labeled clinical datasets	Federated and generative learning strategies
Interpretability	Unstable post hoc explanations	Clinically validated and causal XAI
Multimodal fusion	Missing or noisy modalities	Adaptive, uncertainty-aware fusion models
Computational efficiency	High training and inference cost	Green and lightweight AI architectures
Clinical validation	Predominantly retrospective studies	Prospective and real-world AI trials

## Data Availability

No new data were created or analyzed in this study.
